# Preparation of Deuterium-Labeled Armodafinil by Hydrogen–Deuterium Exchange and Its Application in Quantitative Analysis by LC-MS

**DOI:** 10.3390/metabo12070578

**Published:** 2022-06-22

**Authors:** Paulina Grocholska, Robert Wieczorek, Remigiusz Bąchor

**Affiliations:** Faculty of Chemistry, University of Wroclaw, F. Joliot-Curie 14, 50-383 Wroclaw, Poland; paulina.grocholska@chem.uni.wroc.pl (P.G.); robert.wieczorek@chem.uni.wroc.pl (R.W.)

**Keywords:** LC-MS, armodafinil, internal standards, hydrogen–deuterium exchange, HDX, isotope dilution

## Abstract

Armodafinil, the R enantiomer of modafinil, was approved in 2007 by the US Food and Drug Administration as a wake-promoting agent for excessive sleepiness treatment. Due to its abuse by students and athletes, there is a need of its quantification. Quantitative analysis by liquid chromatography-mass spectrometry, however, though very common and sensitive, frequently cannot be performed without isotopically labeled standards which usually have to be specially synthesized. Here we reported our investigation on the preparation of deuterated standard of armodafinil based on the simple and inexpensive hydrogen–deuterium exchange reaction at the carbon centers. The obtained results clearly indicate the possibility of introduction of three deuterons into the armodafinil molecule. The introduced deuterons do not undergo back exchange under neutral and acidic conditions. Moreover, the deuterated and non-deuterated armodafinil isotopologues revealed co-elution during the chromatographic analysis. The ability to control the degree of deuteration using different reaction conditions was determined. The proposed method of deuterated armodafinil standard preparation is rapid, cost-efficient and may be successfully used in its quantitative analysis by LC-MS.

## 1. Introduction

Armodafinil (ARM, (-)-2-[(*R*)-(diphenylmethyl) sulfinyl] acetamide), is one of the enantiomers of modafinil (MOD) [1]. Both compounds are used to treat sleep disorders, including narcolepsy, sleep apnea, and shift work sleep disorders. Moreover, the main role of armodafinil is to stimulate the central nervous system and interfere with the daily rhythms of sleep and wakefulness, hence it belongs to the group of compounds called eugeroics. The mechanism of action of modafinil has not been clearly established. However, it may indirectly increase wakefulness, at least in part, through inhibition of cortical γ-aminobutyric acid (GABA) release via serotonergic mechanisms [2]. Unfortunately, these chemicals have some disadvantages such as addiction; however, they are less addictive than, for example, cocaine, which also binds to the dopamine transporter [3,4,5]. Drug Rating Questionnaire from the work of Jasinski [6] have reported that 800 mg of modafinil was similar to 90 mg of methylphenidate.

Due to the cases of abuse and side effects, armodafinil and modafinil may cause, some quantitative and qualitative analytical techniques have been proposed in the literature data [7]. The selective capillary electrophoresis (CE) method using sulfobutyl ether-β-cyclodextrin as chiral selector has been developed and validated for the determination of enantiomeric impurities with *R*-modafinil, i.e., armodafinil [8]. Several studies on the enantioseparation and quantification of modafinil on a biological matrix or formulation have been reported [9,10,11,12,13]. The limit of detection (1.25 µg/mL) and the limit of quantification (2.50 µg/mL) were also determined [8]. However, most of these methods are quite expensive because of the costs of chiral columns. So far, no quality control studies have been identified for *S*-modafinil.

The HPLC methods have also been developed for the analysis of modafinil and its metabolites in plasma and urine [9,14,15,16]. These methods have been mainly used for the pharmacokinetic studies of modafinil and its enantiomers. The HPLC method can be used to screen plasma and urine samples for modafinil, and for the pharmacokinetic studies as well as for therapeutic monitoring. Recently, liquid chromatography-mass spectrometry (LC-MS) has become the leading technique in the analysis of drugs, their metabolites and biomolecules especially due to its sensitivity, selectivity and quickness. The quantification of various chemicals, using mass spectrometry, requires application of isotopically labeled standards. Their preparation is usually expensive and requires de novo synthesis of a given compound. The applied isotopologues, containing ^13^C, ^15^N, ^18^O or ^2^H isotopes, should present identical chromatographic behavior to the native compounds, and must be distinguishable by mass shift at least of 2 Da. Additionally, such compounds cannot undergo back-exchange under the LC-MS separation conditions [17]. The introduction of deuterium, although considered cheap, carries the risk of an isotope effect, associated with the lack of isotopologues co-election of [18].

In 2018, Chandasan et al. [19] developed a method of armodafinil quantification in human plasma based on the application of its commercially available standard, armodafinil-d_10_ and LC-MS technique. The proposed strategy allows for armodafinil quantification in the range of 10–10,000 ng/mL, with the LLOQ of 10 ng/mL, much lower than previously developed methods [20]. Schwertner and Kong [21] developed a procedure for the quantitative analysis of modafinil in urine and plasma using reverse phase high-performance liquid chromatography. The lower limit of detection was 0.1 µg/mL [21]. Moreover, it can be easily adapted to the LC-MS analysis of armodafinil [21]. The limitation of these methods is the application of expensive, commercially available deuterated standard of armodafinil. Therefore, there is a strong need to develop more effective and cost-efficient methods of isotopically-labeled standards.

Recently, we reported our investigation on the hydrogen–deuterium exchange of carbon-bounded hydrogen atoms in peptides containing *N*-substituted glycine and alanine residues, via base-catalyzed hydrogen–deuterium exchange [22,23,24,25]. This observation allowed us to developed methods of deuterated standards preparation of denatonium benzoate [26], cyclosporine A [27] and creatinine [28], which were successfully applied in their quantification by LC-MS. We found that the introduced deuterons do not undergo back-exchange either in an acidic or neutral conditions and that the isotopologues co-elute.

In armodafinil molecule the sulfinylacetamide moiety, a structure similar to previously investigated *N*-substituted glycine derivatives, where the slightly acidic hydrogen atoms on the α-carbon are presented. Therefore, the main goal of our study was to investigate the possibility of hydrogen–deuterium exchange of carbon-bound hydrogens within the armodafinil molecule and to test the possibility of application of the obtained deuterated standard in the quantitative analysis by LC-MS. According to the obtained data, the proposed method reduces the cost of deuterated armodafinil preparation at least 100 times.

## 2. Results

### 2.1. Analysis of the Hydrogen–Deuterium Exchange at the Carbon Atoms

**Figure 1:**
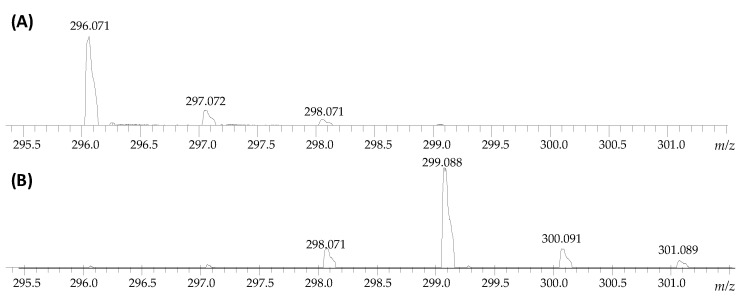
ESI-MS analysis presenting isotope pattern of sodium adduct of armodafinil ion (**A**) before HDX and (**B**) after incubation in the mixture of 1% TEA/D_2_O/MeCN for 3 days in the room temperature.

The aim of this work was to obtain a deuterated armodafinil derivative as an isotopically-labeled standard for quantitative analysis by mass spectrometry and its full characterization by tandem mass spectrometry, liquid chromatography and nuclear magnetic resonance, as well as to perform a quantitative analysis of armodafinil in biological samples. Based on our previous investigation [22,23,26], we applied base-catalyzed hydrogen–deuterium exchange to investigate the possibility of armodafinil deuteration at the carbon atoms. The 1% solution of *N,N,N*-triethylamine (TEA) in the mixture of D_2_O/MeCN was used. The sample was incubated from 1 to 7 days at room temperature. Then the samples were lyophilized and redissolved in the mixture of H_2_O/MeCN/HCOOH (50/50/0.1 *v*/*v*/*v*) and the progress of armodafinil deuteration was monitored by ESI-MS. It was observed that even after 3 days of incubation of armodafinil in the 1% TEA/D_2_O/MeCN mixture in the room temperature the most intensive signal within the isotope pattern was shifted by 3 Da, corresponds to the exchange of three hydrogen atoms to deuterons (Figure 1).

**Figure 2:**
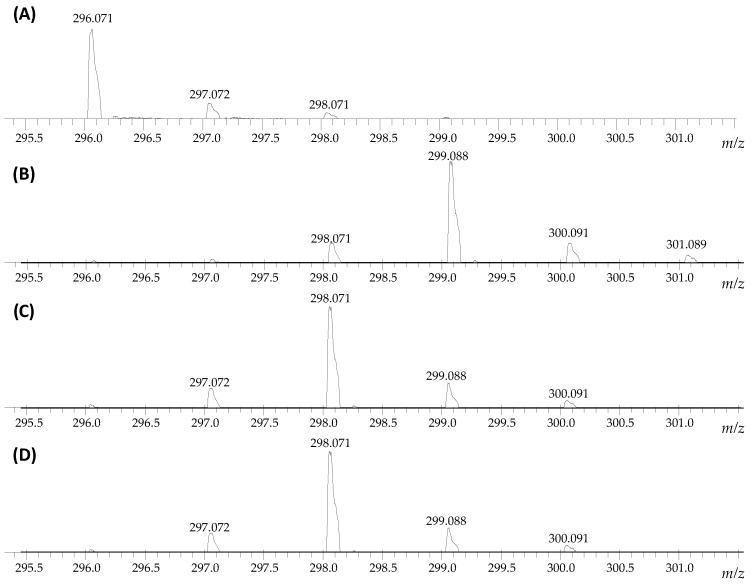
Fragment of the ESI-MS spectra presenting isotope pattern of sodium adduct of armodafinil ion (**A**) before HDX and after 1 h incubation of the sample at 50 °C in the mixture of (**B**) 1% TEA/D_2_O/MeCN, (**C**) 1% TEA/MeOD, (**D**) 1% TEA/MeOD/TBME, lyophilization and treated by the mixture of H_2_O/MeCN/HCOOH (50/50/0.1 *v*/*v*/*v*).

Longer incubation times did not provide any changes in the isotope pattern, therefore, we decided to optimize the HDX conditions. Based on the obtained results (Figure 1), the reaction time was significantly shortened and the influence of the temperature and solvent on the progress of armodafinil deuteration were investigated. Because the armodafinil is soluble in methanol and *tert*-butyl methyl ether (TBME), we decided to perform the hydrogen–deuterium exchange reaction in these solvents. In both cases, deuterated methanol (Me-OD) was used as a source of deuterons. The samples were incubated both in the room temperature and at 50 °C for 1 h and 3 h. The obtained samples were lyophilized, redissolved in the mixture of H_2_O/MeCN/HCOOH (50/50/0.1 *v*/*v*/*v*) and analyzed by ESI-MS to monitor the progress of hydrogen–deuterium exchange. The obtained results showing the highest degree of armodafinil deuteration are presented in the Figure 2.

**Figure 3:**
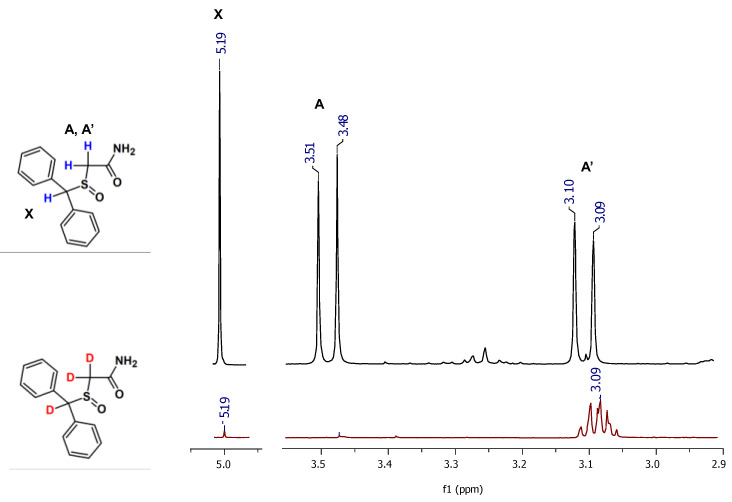
^1^H NMR spectra of armodafinil (black line) and armodafinil-d_3_ (red line) samples dissolved in CDCl_3_.

On the obtained mass spectra, the signal corresponding to the sodium adduct of armodafinil-d*_3_* was found even after 1 h incubation in the mixture of 1% TEA/D_2_O/MeCN in the room temperature (Figure 2B). Some unexchanged fractions remained (Figure 2B, 298.071 *m*/*z*). After the same time of incubation in the mixture of 1% TEA/MeOD (Figure 2C) and 1% TEA/MeOD/TBME (Figure 2D), the signal characterizes doubly deuterated armodafinil analogue (298.071 *m*/*z*), was observed. Based on the results obtained by ESI-MS method, we performed the ^1^H-NMR analysis of the armodafinil sample presenting the highest degree of deuteration. The obtained results for the samples incubated in the mixture of 1% TEA/D_2_O/MeCN for 1 h at 50 °C are presented in the Figure 3.

The presented ^1^H-NMR spectra (Figure 3) suggest that, after 1 h of HDX reaction, the signal at 5.20 ppm, characterizing proton X, practically disappeared. The signals corresponding to the α-C hydrogens (A, A’) at 3.50 ppm and 3.10 ppm become a multiplet, which may suggest that a small amount of armodafinil is not completely deuterated and the signal for α-CHD group is observed. The incomplete deuteration was also present on the obtained mass spectra (Figure 1 and Figure 2), where the small signal corresponding to the 297.072 *m*/*z* was presented.

### 2.2. Fragmentation of Deuterated and Non-Deuterated Armodafinil

**Figure 4:**
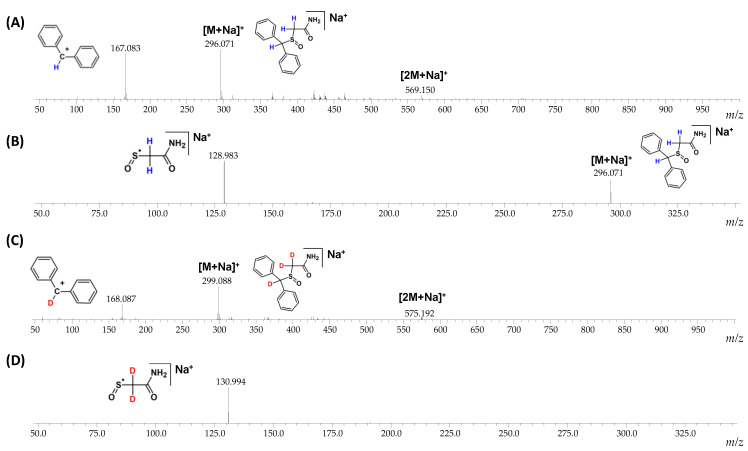
(**A**) ESI-MS spectrum of armodafinil, (**B**) ESI-MS/MS spectrum of armodafinil—parent ion 296.071 *m*/*z*, collision energy 30 eV, (**C**) ESI-MS spectrum of armodafinil-d_3_, (**D**) ESI-MS/MS spectrum of armodafinil-d_3_—parent ion 299.088 *m*/*z*, collision energy 30 eV.

**Figure 5:**
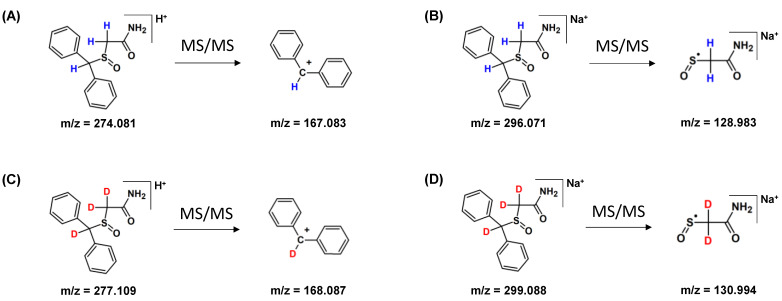
Fragmentation pathways of (**A**) protonated armodafinil, (**B**) armodafinil sodium adduct, (**C**) protonated armodafinil-d_3_, (**D**) sodium adduct of armodafinil-d_3_.

The fragmentation pathways of armodafinil and its deuterated analogue was analyzed by ESI-MS/MS (Figure 4). In both cases the signal characterized sodium adduct of armodafinil was observed at the 296.071 *m*/*z* and 299.088 *m*/*z*, for native and deuterated analogue respectively. On the obtained ESI-MS spectra, the characteristic formation of stable secondary aromatic carbocation at 167.083 *m*/*z* (Figure 4A) for non-deuterated armodafinil and at 168.087 *m*/*z* for armodafinil-d_3_ was observed (Figure 4C). In the MS/MS mode, the sodium adduct of armodafinil and armodafinil-d_3_ analogue forms the aliphatic radical coordinating sodium ion at 128.983 *m*/*z* (Figure 4B) and 130.994 *m*/*z* (Figure 4D), respectively. The fragmentation pathways of protonated armodafinil ion and its sodium adduct are schematically presented in the Figure 5.

### 2.3. IR Analysis

**Figure 6:**
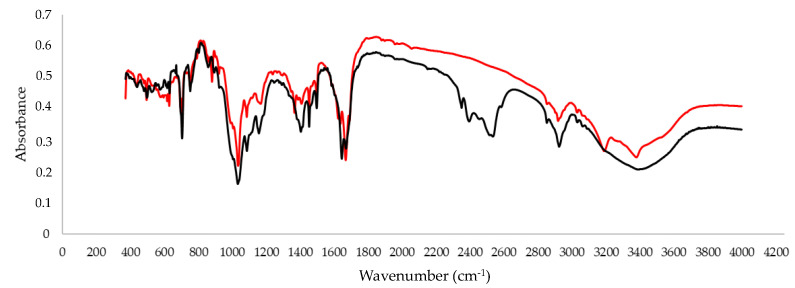
IR spectra of armodafinil (red line) and armodafinil-d_3_ (black line) in KBr.

We also performed the infrared spectroscopy analysis for the d_3_ standard of armodafinil. The characteristic additional bands at 2400–2600 cm^−1^ (Figure 6), related to the C-D bonds, were observed.

### 2.4. Computational Analysis of Deprotonation Properties of Armodafinil

In order to determine the deprotonation properties of investigated molecules, quantum chemical calculations were performed at the DFT (density functional theory) as experimentally verified tool for investigation of structure and properties of complexes and molecules [29,30,31,32,33]. To determine the order of the proton dissociation, we have calculated the enthalpies of the reactions of proton disconnection at 298 K (∆H_298_) from an inert neutral molecule in accordance with reaction:

molecule^0^ + H_2_O→molecule^−^ + H_3_O^+^

**Figure 7:**
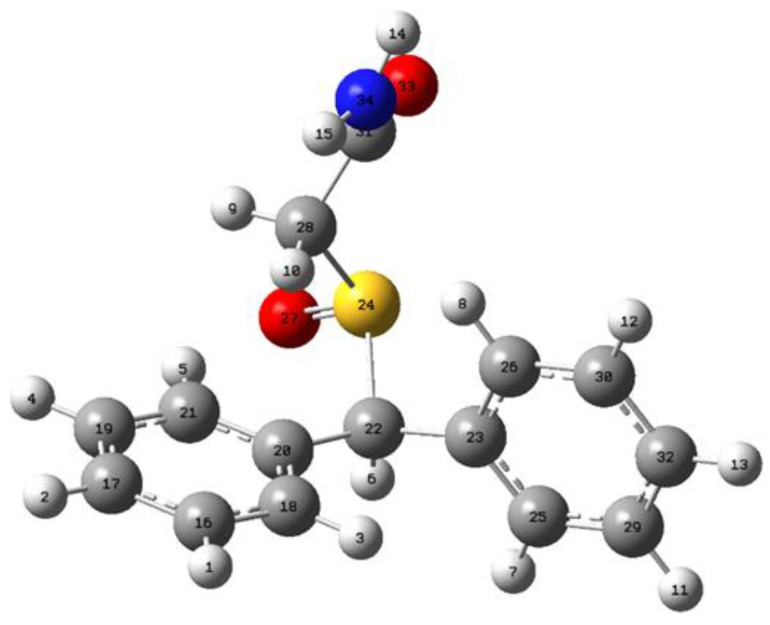
The structure and deprotonation positions of the armodafinil.

The deprotonation of armodafinil can be achieved in one of the fourteen ways, shown with the numbered (1–14) hydrogen atoms in Figure 7.

As one could expect, the values ∆H^298^ in Table 1 indicate that in order for proton disconnection, the least energy shall be provided when the C22 atom is deprotonated.

**Figure 8:**
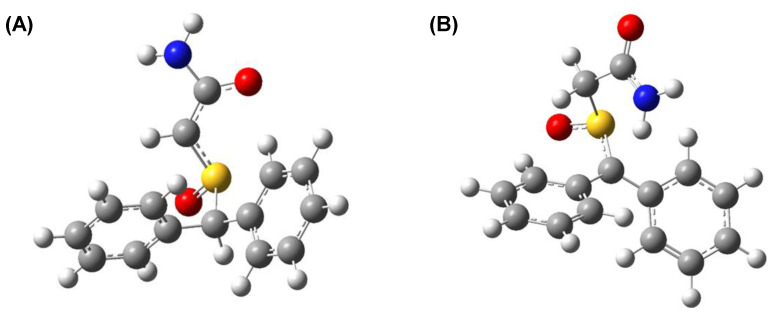
The structure of deprotonated armodafinil (**A**) at the C28 atom and (**B**) at the C22.

Please note that next deprotonation position is located on amino group with the energy gap only ~3 kcal/mol. The structure of the deprotonated armodafinil at the C22 and C28 atoms is presented in the Figure 8. The deprotonation in C22 position causes electron density redistribution. The most significant change in atom charges we observe are on C22, which shows −0.2 charge difference in comparison to the neutral molecule. Similarly to deprotonation in C22, the C28 position deprotonation brings the most significant changes in the deprotonated atom area. The C22 atom charge changes by −0.2 in comparison to the neutral molecule.

C22 atom is located within the sulfinylacetamide moiety. The hydrogens in this position are surrounded by electron-withdrawing groups, especially the S=O group, where the inductive negative effect of the oxygen atom reduces the electron density on the carbon–hydrogen bonds, making them more acidic. The more acidic the hydrogen atoms are, the easier it is to exchange them to deuterons, especially in the base-catalyzed reaction. Additionally, the presence of both, amide and S=O group in the neighborhood of C22 hydrogens stabilize the adjacent carbanion, formed as an intermediate during the HDX reaction, by the resonance.

C28 atom has one hydrogen atom. The proximity of S=O group, having a negative inductive effect, lower the electron density in the C-H bond which increase its acidity. Moreover, the benzylic position is quite reactive and presents a useful synthetic tool for preparing many aromatic compounds. It results from the resonance stabilization of the benzylic carbon whether the ionic or radical mechanism take place.

### 2.5. Deuterium–Hydrogen Exchange Analysis

**Figure 9:**
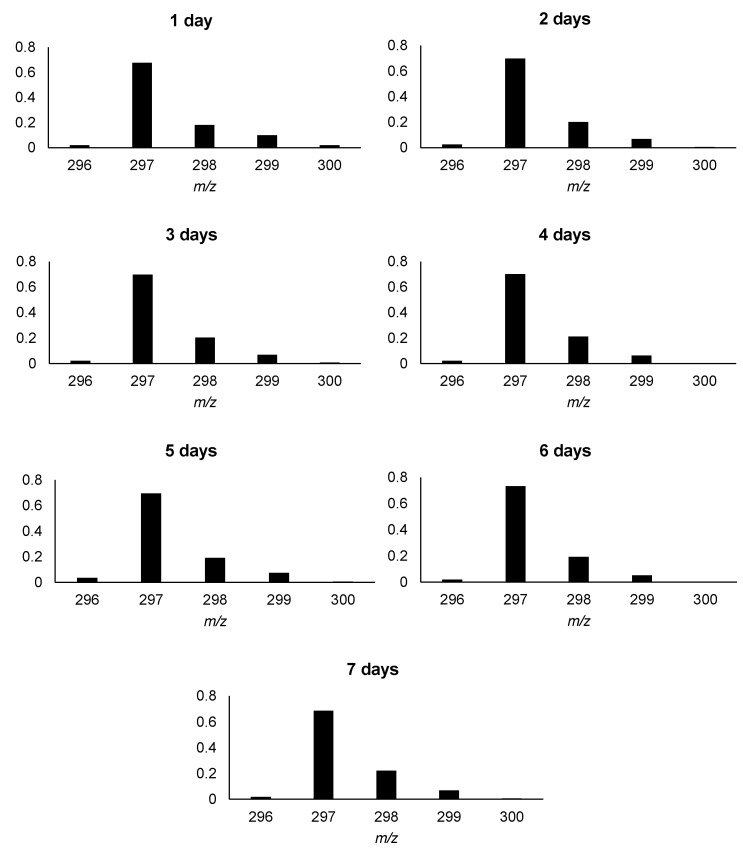
Kinetics of DHX reaction of armodafinil-d_3_ in 1% TEA/H_2_O/MeCN/solutions.

Hydrogen–deuterium exchange and deuterium–hydrogen exchange (DHX) usually present the same kinetics with some differences depending on the possibility of hydrogen- or deuterium bonds formation and the spatial structure of chemical compounds [34,35]. Therefore, the analysis of the DHX kinetics on the arodafinil-d_3_ isotopologue was performed for the obtained armodafinil-d_3_ isotopologue and the results are presented as normalized values of signal intensities from ESI-MS isotope pattern (Figure 9). The separate armodafinil-d_3_ samples were incubated in the mixture of 1% TEA/H_2_O/MeCN from 1 to 7 days. The obtained results revealed that in all cases the most intensive signal at 297.072 *m*/*z*, corresponding to the armodafinil derivative containing one unexchanged deuteron (Figure 9).

**Figure 10:**
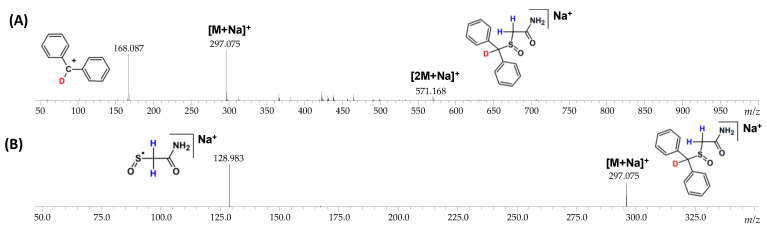
(**A**) ESI-MS and (**B**) MS/MS (parent ion 297.075 *m*/*z*, collision energy 30 eV) spectra of armodafinil-d_1_ obtained after deuterium–hydrogen exchange.

To determine the location of the remained deuterium atom after the DHX, the MS/MS experiments were performed, and the obtained spectra are presented in the Figure 10. The ESI-MS spectrum of armodafinil-d_1_ obtained after the analysis of DHX presents intensive signal at *m*/*z* 168.087 corresponding to the secondary carbocation containing deuterium atom (Figure 10A). The MS/MS spectrum obtained for parent ion at *m*/*z* 297.075 shows the signal corresponding to the aliphatic radical coordinating sodium ion at *m*/*z* 128.983 without any deuterium atom (Figure 10B). The obtained results clearly indicate that the unexchanged deuterium atom remained at the carbon atom within the diphenylmethyl group.

### 2.6. Stability of Deuterated Armodafinil

**Figure 11:**
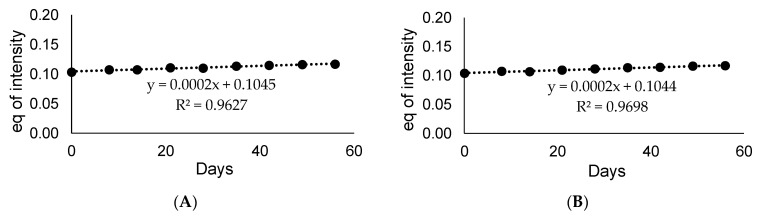
Stability curve of (**A**) non-deuterated armodafinil and (**B**) armodafinil-d_3_ measured till two months for the ion (**A**) 296.071 *m*/*z* and (**B**) 299.088 *m*/*z*.

To apply the obtained deuterated standard in the quantitative analysis using LC-MS, the introduced deuterons should be stable under LC-MS conditions and do not undergo back-exchange [20]. We analyzed the stability of both isotopologues under solution using in MS analysis, which is water and acetonitrile with addition of 0.1% of formic acid (1 mL of H_2_O/MeCN/HCOOH 1:1:0.1 *v*/*v*/*v*), in the room temperature, and then analyzed by LC-MS method. The obtained results are presented in the Figure 11. It was found, that even after 60 days of incubation of armodafinil-d_3_ in the mixture of H_2_O/MeCN/HCOOH (1:1:0.1 *v*/*v*/*v*), the introduced deuterons did not undergo back-exchange and no decomposition reaction took place.

### 2.7. Quantitative LC-MS Analysis

**Figure 12:**
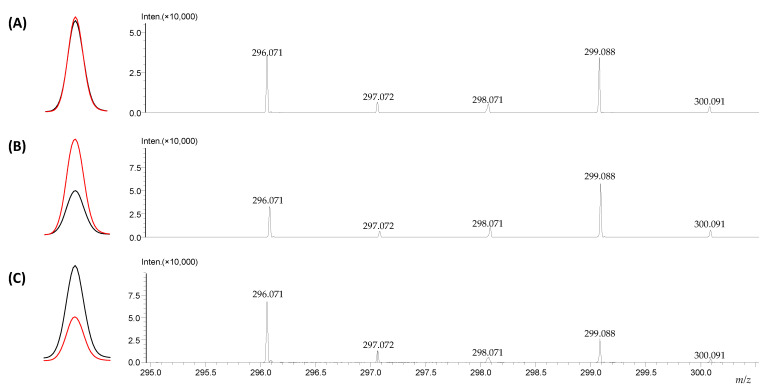
Extracted ion chromatograms and corresponding mass spectra of the analyzed samples containing armodafinil (black line) and armodafinil-d_3_ (pink line) mixed in (**A**) 1:1 ratio (**B**) 1:2 ratio, (**C**) 2:1 ratio.

One of the requirements for isotopically labeled standards, in addition to their stability under the conditions of the analysis, considered as lack of back exchange of the introduced isotopes, is the co-eluction of the deuterated and non-deuterated isotopologues which may be affected by the isotopic effect [20,36]. To test the co-elution, armodafinil and its deuterated d_3_ analogue were mixed in 1:1, 1:2 and 2:1 ratio. The obtained results are presented in the Figure 12.

The presented extracted ion chromatograms (Figure 12) of the armodafinil (black line) and armodafinil-d_3_ (red line) isotopologues presents practically the same chromatographic behavior for all the analyzed samples containing different amounts of isotopologues (Figure 12A–C). The proportion of signals corresponding to the armodafinil and armodafinil-d_3_ analogues to their amount in the analyzed mixtures was confirmed. Additionally, the isotopic distribution presented on the obtained mass spectra clearly corresponds to the composition of the analyzed mixtures.

**Figure 13:**
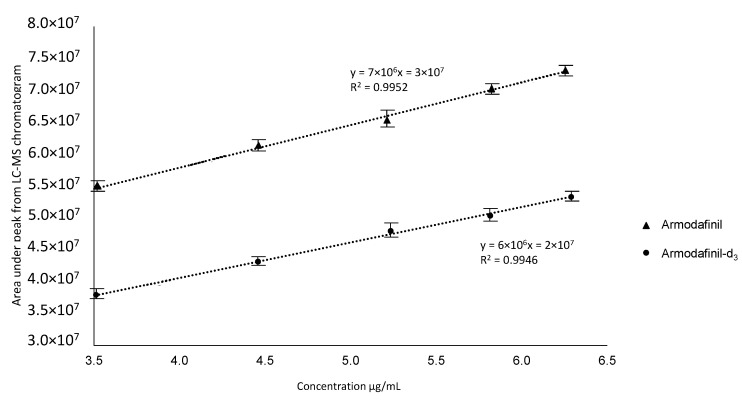
Calibration curve of non-deuterated armodafinil and armodafinil-d_3_.

To perform the quantitative analysis of the armodafinil by LC-MS, we prepared curve for both armodafinil isotopologues (Figure 13). This curve was constructed by plotting the peak area ratio of each compound (y) versus the concentration of the compound (x, µg/mL).

**Figure 14:**
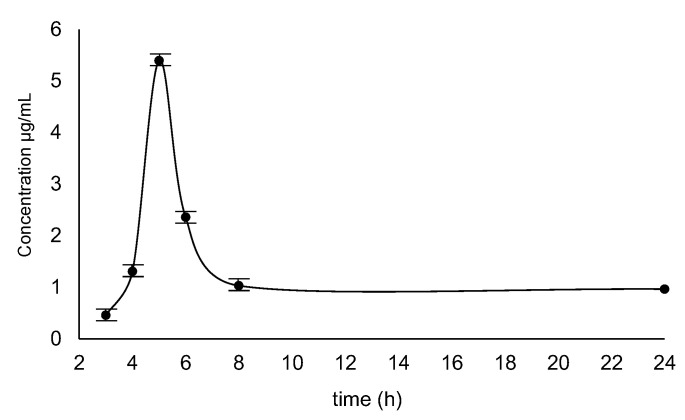
Concentration curve of armodafinil in human urine samples collected after 3 h, 4 h, 5 h, 6 h, 8 h and 24 h after taking 200 mg dose.

The human urine samples collected after 3, 4, 5, 6, 8 and 24 h after taking 200 mg of armodafinil by one patient were analyzed. The obtained results are presented in the Figure 14. The maximum concentration of armodafinil after single-dose was found in the urine sample collected after 5 h of its taking, at the level of 5.3 µg/mL. It was observed that armodafinil is readily absorbed after oral administration and also readily eliminated. After reaching peak, urine concentration of armodafinil decreases relatively quickly. The obtained results correspond to the data presented previously by two clinical teams [37,38].

The matrix effect was investigated. The armodafinil solutions with the concentration of 3.5, 4.5, 5.3, 5.9 and 6.4 µg/mL were performed by the dilution of stock solution (1 mg/mL in H_2_O/MeCN/HCOOH mixture (50/50/0.1 *v*/*v*/*v*)). During the analysis of matrix effect, the appropriate volume of stock solution was dissolved in urine sample. The obtained samples were analyzed by LC-MS. The obtained data present that the armodafinil was detected at the level of 10 ng/mL when prepared in the H_2_O/MeCN/HCOOH mixture. The presence of urine as matrix resulted in a slight decrease in the detection level to 100 ng/mL.

## 3. Discussion

Hydrogen–deuterium exchange involves the hydrogen atom substitution by a deuterium atom in a molecule, in the presence of a source of deuterons, e.g., deuterium oxide (D_2_O), deuterated methanol (MeOD) or another source [39]. The hydrogen atoms present in the functional groups of chemical compounds are exchangeable with the solvent protons within the minutes and the mechanism of this process includes the acid–base catalysis where the reaction rate strongly increases with increasing pH [40]. The possibility of HDX of carbon-bound hydrogen atoms was also investigated [41]. The hydrogens attached to carbons are mostly not exchanges by the deuterons; however, the pH-dependent and metal-dependent catalysis may allow for such transformation [25]. The carbon-bound hydrogen atoms are characterized by different acidity being a consequence of their distinct chemical environment, where the presence of electron-withdrawing groups plays a key role in the discussed HDX [42,43,44].

Sulfinylacetamide moiety (Figure 2), is a structure which can be considered as similar to glycine fragment with hydrogens on the α-carbon atom, surrounded by electron-withdrawing groups. As presented by Rios and co-workers [44], the acidity of hydrogens located at the alpha-carbon atom within the glycine moiety depends on the ionization state of the amino acid and substitution of the amino group where the exhaustive *N*-methylation also affects the pKa of discussed hydrogens. The sulfinylacetamide moiety may activate the α-hydrogen as a consequence of the negative inductive effect of S=O group, and stabilize the adjacent carbanion by the resonance (Figure 8A) [45]. Additionally, the diphenylmethyl group hydrogen, due to the presence of S=O group, can undergo hydrogen–deuterium exchange due to the resonance stabilization of the phenyl rings (Figure 8B). Rios and co-workers [44] proposed explanation of the observed hydrogen–deuterium exchange at α-C assumes that the observed phenomenon is a consequence of combination of inductive and resonance stabilization of the corresponding enolates. Based on the computational analysis, which we performed, it was found that the deprotonation of both carbon atoms causes electron density redistribution, leading to the formation of resonance stabilized structures (Figure 8A,B).

In our investigation we observed that the hydrogen–deuterium exchange of the carbon-bound hydrogens within the sulfinylacetamide moiety occur faster than in the case of previously analyzed by us *N*-methylated glycine derivatives [22,23,26]. This observation confirms higher acidity of the carbon-bound hydrogen atoms within the armodafinil molecule, as based on the kinetics of the analyzed process (Figure 1, Figure 2 and Figure 3).

The exchange of hydrogen to deuterium may cause the isotope effect, affecting the isotopologues co-elution during chromatographic analysis. As a result of the larger mass of deuterium, the amplitude of C-D bond vibrations is smaller than for C-H bond, resulting in slightly lower average volumes and polarizabilities for bonds involving deuterium [46,47]. In this phenomenon, the number of the introduced deuterons and their location play a crucial role [18]. It was found that the deuterium effect is lower when the deuterons are introduced in the polar part of the molecule [18,25]. Within the armodafinil molecule, the deuterons are located in the polar part of the molecule, which clearly explain our observation of the lack of isotopic effect and isotopologues co-elution during their chromatographic separation (Figure 12).

During the MS and MS/MS analysis of the deuterated and non-deuterated analogues of armodafinil, the different fragment ions were found (Figure 4, Figure 5 and Figure 10). In the ESI-MS analysis (Figure 4A,C), the high intense signal at 167.087 (Figure 4A) and at 168.087 (Figure 4C), characterizing the diphenylmethylium carbocation was observed. Additionally, the signal corresponding to the protonated armodafinil ion was not observed. The lack of observation of the [M+H]+ ion is a consequence of its low stability in the ion source, leading to the formation of diphenylmethylium according to the charge-directed mechanism [34]. Additionally, in the MS/MS spectra (Figure 4B,D), the fragmentation of sodium adduct of armodafinil ([M+Na]^+^), resulted in the formation of (2-amino-2-oxoethyl)(oxo)-sulfanyl radical. This observation is a consequence of the charge-remote fragmentation mechanism of [M+Na]^+^ ion of armodafinil [48].

As shown in Figure 9, deuterium–hydrogen exchange presents different isotope patterns than the HDX of carbon-bound hydrogen atom in armodafinil molecule (Figure 2). This phenomenon becomes visible by a lack of the signal corresponding to the non-deuterated [M+Na]^+^ armodafinil ion at 296.071 *m*/*z*. The reduced exchange efficiency can be ascribed only to the different properties of C-D bond, where the mass difference between hydrogen and deuterium affects the vibrational energy within C-H and C-D bonds, resulting in the slower oscillation of the bond. According to the Giagou and Meyer [49], the kinetic isotope effect is a tool that allow to measure the extent to which changes in isotopic distribution at a given position in a reactant molecule affect the rate of the reaction. The zero-point energy for the C-H bond is significantly higher than for the C-D bond, and the average length of C-H bond is greater than that for C-D bond [50]. The smaller reduced mass associated with C-H stretching vibrations translates into a more disperse wave-function. Conceptually, this can be thought of in terms of the lighter particle having more wavelike character than the heavier particle. These factors contribute to our observation where under the applied reaction conditions, HDX and DHX experiments delivered non-mirrored results.

The proposed method of the armodafinil analysis by LC-MS revealed the same level of detection and quantification as previously presented [19,20,21]; however, the most important advantage of the developed strategy is the low-cost preparation of the deuterated armodafinil standard reducing the cost of it costs at least 100 times, which can make it much more profitable and widely used.

## 4. Materials and Methods

### 4.1. Reagents

Armodafinil, deuterium water (D_2_O, 99.9% purity), methanol—d_1_ (MeOD, 99.9% purity), *t*-butylmethyl ether (TBME), *N,N,N*-triethylamine (TEA), acetonitrile (MeCN), formic acid (HCOOH) were purchased from Sigma-Aldrich (Saint Louis, MO, USA). The used solvents were all of LC-MS grade.

### 4.2. Mass Spectrometry

The ESI-MS experiments were all performed on a LCMS-9030 qTOF Shimadzu (Shimadzu, Kyoto, Japan) device, equipped with a standard ESI source and the Nexera X2 system. The analysis was performed in the positive ion mode between 50 and 2000 *m*/*z*. The LCMS-9030 parameters were as follows: nebulizing gas, nitrogen; nebulizing gas flow, 3.0 L/min; drying gas flow, 10 L/min; heating gas flow, 10 L/min; interface temperature, 300 °C; desolvation line temperature, 400 °C; detector voltage, 2.02 kV; interface voltage, 4.0 kV; collision gas, argon; collision energy was optimized between 10 and 30 eV. The injection volume was 0.1 µL. Analyte solutions were introduced at a flow rate of 0.3 µL/min in a H_2_O/MeCN mixture (50:50, *v:v*). The signals obtained on the mass spectra had all a mass accuracy error in the range of 1 ppm. The chromatographic module was operated as follows: eluent (A) water + 0.1% HCOOH, eluent (B) acetonitrile + 0.1% HCOOH. The obtained data were analyzed by LabSolutions 4.0 software (Shimadzu, Kyoto, Japan).

### 4.3. CID

The singly charged precursor ions were selected on the quadrupole and subsequently fragmented in the hexapole collision cell. A very narrow isolation window (±0.1 Da) was used for selective isolation of one signal. Argon was used as a collision gas. The obtained fragments were registered as an MS/MS (tandem MS) spectrum. The collision voltage (15–30 V) was optimized for the best fragmentation. For MS analysis, a LabSolutions software was used.

### 4.4. Isotopic Exchange

HDX was performed by dissolving 2.0 mg of the armodafinil in the mixture of 500 µL MeOD/TBME (1:1, *v:v*) at room temperature. The sample was mixed and incubated at 30 °C for 1h and lyophilized. The sample was redissolved in 200 µL of H_2_O/MeCN (1:1, *v*/*v*) mixture, incubated for 30 min and subjected to an ESI-MS analysis. In order to estimate the pD of the analyzed alkaline solutions, the pH was measured using a MP230 pH meter (Mettler-Toledo, Greifensee, Switzerland). The pD was calculated according to the equation: pD = pH + 0.4 [51]. The obtained pD value was 12.4 for 1% TEA/D_2_O mixture at room temperature.

DHX was performed by redissolving the armodafinil-d_3_, which was obtained by the method described earlier, in the mixture of 500 µL 1% TEA/H_2_O/MeCN (1:1:0.1, *v*:*v*:*v*) at room temperature. The sample was mixed and incubated at 50 °C for 1h and lyophilized. The solid was redissolved in 200 µL of a H_2_O/MeCN (1:1, *v*/*v*) mixture, incubated for 30 min and analyzed by ESI-MS analysis.

### 4.5. H-NMR and ^13^C-NMR Analysis

NMR spectra were recorded on a high-field Bruker Avance 500 MHz NMR spectrometer (Bruker Daltonics, Bremen, Germany). A ^1^H-NMR and ^13^C-NMR analysis were performed for each armodafinil sample after HDX. Samples were dissolved in CDCl_3_.

### 4.6. IR Spectroscopy

FTIR spectra were recorded between 4000–400 cm^−1^ in a transmission mode by means of a Bruker IFS 66/S FTIR spectrometer with a resolution of 0.5 cm^−1^ and using a liquid N2 cooled MCT detector. Matrices were irradiated with the tunable UV light provided by the frequency doubled signal beam of a pulsed (7 ns) optical parametric oscillator Vibrant 355 (Opotek Inc., Carlsbad, CA, USA) pumped with a pulsed Nd:YAG laser (Quantel, Edinburgh, UK). The experiments started using λ  =  370 nm light and proceeded with gradual decrease in the output wavelength. After each irradiation, an infrared spectrum of the matrix was taken.

### 4.7. Computational Methods

All calculations were performed at the DFT (density functional theory) using ωB97X-D [52] long-range corrected hybrid density functional with damped atom–atom dispersion corrections as well describing both the structure and energy of organic compounds [53,54]. The functional triple-ζ basis set 6-311G(d,p) containing polarization functions, was chosen for the calculations.

All the presented structures were fully optimized with demanding convergence criteria (RMS Force = 1 × 10^−5^, RMS Displacement = 4 × 10^−5^, Max Force = 2 × 10^−5^, Max Displacement = 6 × 10^−5^) predefined as “opt = tight” in the Gaussian packet, in atomic units. Calculations were performed with Gaussian 09 rev. E01 [55] software, molecules drawings were made with GaussView 5.08 [56].

To determine the order of the proton dissociation, we have calculated the enthalpies of the reactions of proton disconnection at 298K (∆H298) from an inert neutral molecule in accordance with reaction:

molecule^0^ + H_2_O→molecule^−^ + H_3_O^+^

We have calculated all possible proton dissociation with full range of protons from 10 up to 21 depending on molecule.

### 4.8. Liquid Chromatography-Mass Spectrometry (LC-MS) Analysis

LC-MS experiments were performed on a LCMS-9030 qTOF Shimadzu (Shimadzu, Kyoto, Japan) device, equipped with a standard ESI source and the Nexera X2 system, equipped with an Aeris Peptide XB-C18 column (50 mm × 2.1 mm) with a 3.6 µm bead diameter equilibrated at 27 °C. The LC system was operated with the following mobile phases, eluent (A) water + 0.1% HCOOH, eluent (B) acetonitrile + 0.1% HCOOH, the gradient conditions (B%) were from 40% to 70% B within 17 min. The flow rate was 0.1 mL/min and the injection volume 0.1 µL. For data analysis, LabSolutions software, version 4.0 was used.

## 5. Conclusions

In conclusion, we demonstrated the possibility of preparation of deuterated armodafinil standard via base-catalyzed hydrogen–deuterium exchange at the carbon atom. The introduced three deuterons do not undergo back-exchange under neutral and acidic conditions, and the deuterated form co-elute with the non-deuterated one. The applicability of the obtained armodafinil-d_3_ standard in the quantitative analysis of armodafinil in human urine samples by LC-MS was confirmed. The obtained data are in agreement with the known pharmacokinetic profile of armodafinil. Additionally, the analysis of back-exchange revealed the presence of kinetic isotope effect becoming visible by lack of complete exchange of one deuterium atom to hydrogen which may be explained by the differences in C-H and C-D bond lengths and zero-point energies. The developed method of armodafinil-d_3_ standard preparation is simple, rapid and cost-efficient, which make it a novel tool for researchers, clinicians and forensic scientists working on the improvement of diagnostic accuracy and quantitative forensic LC-MS investigation armodafinil.

## Figures and Tables

**Table 1:** The H^298^ (in hartree) of armodafinil anions and ∆H^298^ of deprotonation reaction (kcal/mol). The most energetically effective deprotonation position is marked with red font.

Deprotonation	H^298^	∆H^298^
6	−1183.109880	106.69
3	−1183.105261	109.58
9	−1183.104098	110.31
10	−1183.104091	110.32
7	−1183.103049	110.97
15	−1183.102962	111.03
14	−1183.098462	113.85
8	−1183.061218	137.22
5	−1183.058096	139.18
4	−1183.050528	143.93
1	−1183.050000	145.66
12	−1183.047156	146.05
11	−1183.046098	146.71
13	−1183.045805	146.89
2	−1183.044940	147.44

## Data Availability

The data presented in this study are available in the article.
